# Editorial: Environmental, social, and corporate governance and sustainability

**DOI:** 10.3389/fpsyg.2022.1062757

**Published:** 2022-10-24

**Authors:** Taewoo Roh, Minwoo Lee, Byung Il Park

**Affiliations:** ^1^Global Business School, Soonchunhyang University, Asan, South Korea; ^2^Conrad N. Hilton College of Hotel and Restaurant Management, University of Houston, Houston, TX, United States; ^3^College of Business, Hankuk University of Foreign Studies, Seoul, South Korea

**Keywords:** environmental, social, governance, ESG, sustainability, CSR

Welcome to this Research Topic of *Frontiers in Psychology* on Environmental, Social, and Corporate Governance (ESG) and Sustainability. After a thorough blind review procedure, we are pleased to announce that 19 articles have been selected for publication in the Research Topic. We believe that all of these articles address important aspects of ESG in this Research Topic according to the background detailed below.

## Research Topic background

We are currently witnessing dramatic changes in the business environment (Lee et al., 2015), and there is an increasing need for firms to adequately deal with environmental issues, satisfy social expectations, and design appropriate corporate governance. These changes have also been eye-opening for academic researchers and practitioners in terms of the importance of environmental, social, and corporate governance. Specifically, we assume that the global financial crisis, which initially began in the United States in 2008 before spreading to the global economy, functioned as an opportunity to think about the sustainability of corporate investment and its impacts on societies as the crisis directly triggered alterations around the business ecosystem. In other words, the global financial crisis pushed ESG to the forefront of scholarly attention, thus causing it to become a significant practical agenda, and it would not have emerged as rapidly without the catalyst of the crisis. Before 2008, the concept of ESG was not widely known. Although corporate social responsibility (CSR) already existed, the scope of this notion was much narrower. This was due to the fact that firms tended to consider it as part of their strategies and buried themselves in returns through CSR (Hong et al., 2022).

Based on the situations and backgrounds illustrated above, relevant discussions on ESG are still in their infancy, so it is urgent to answer unanswered questions about ESG. For instance, we do not know exactly which factors promote ESG, how ESG differs from CSR, and how ESG contributes to solving social inequalities, and broadly speaking, what macroscopic outcomes can be expected by enforcing ESG. The more serious problem is that, although numerous extant studies (e.g., Khan et al., 2015; Park and Ghauri, 2015) have attempted to dig into CSR, it remains unclear whether the empirical results uncovered by these experiments can be applied to ESG. Moreover, to our knowledge, previous studies that have attempted to link and/or bridge ESG and corporate sustainability are sparse.

Built on this premise, our Research Topic attempts to open up relevant research questions and simultaneously fill in these research gaps. We argue that this Research Topic, which includes the following 19 papers, combines theoretical and empirical refinements linking ESG with various economic actors, such as consumers, businesses, and markets, and that the chosen papers significantly expand our current knowledge on ESG.

## The nineteen papers included in this Research Topic

The first paper by Shaheen et al. addressed how gender-diverse boards in China influence CSR reporting decisions in politically embedded firms and offered fresh insights showing that the presence of female directors and the participation of executives having political connections are highly related to a company's CSR reporting. In improving CSR reporting and helping Chinese corporations better meet their social and environmental goals, the findings of that study can help policymakers develop policy measures that are specifically targeted at legislation regarding women's quotas and the existence of political connections on corporate boards.

The second paper by Kwak et al. analyzed the sensitivity between fund flow and fund performance among Korean funds. The primary findings of the paper are 3-fold: First, ESG did not affect corporate fund flow. Second, the fund flow–performance interaction had a negative correlation. Third, there was almost no difference between the ESG fund sensitivity asymmetry and the non-ESG fund sensitivity asymmetry.

The third paper by Kim et al. was conducted to answer the question, “can ESG activities have significant effects on performance at the subsidiary level?” According to the results of the empirical analyses, among Korean subsidiaries of multinational corporations, ESG activities positively affected firm performance. As a result of examining the moderating effect of market-oriented organizational culture, it was also found that the positive relationship between ESG activities and performance was weakened.

The fourth paper by Bang et al. examined identifying human resource practices coupled with external CSR activities due to the importance of ESG.

The fifth paper by Kim and Lee investigated the effects of recalling COVID-19 concerns on pro-environmental product consumption. The results indicated that consumers exhibited greater purchase intention toward e-prompt products (vs. non-e-prompt products) when they recall altruistic anxieties associated with COVID-19. Considering ESG also mediated the moderating effects. By introducing the nature of COVID-19 fears as a critical factor, this study made a worthwhile contribution to the literature on pro-environmental behavior.

The sixth paper by Lee and Jeong confirmed that the compatibility and authenticity of social responsibility activities affect a firm's brand trust, thereby improving corporate sustainability management. The results of this study provided strategic implications for the performance of social responsibility activities necessary for the continuous growth of firms and for building brand trust.

The seventh paper by Qing and Jin investigated whether CSR can affect sustainability through the economic and social performance of social enterprises (SEs). It also attempted to verify the moderating role of innovativeness in the relationship between CSR and SE performance. The results suggest that, while CSR can improve sustainability through economic and social performance, innovativeness has no moderating effect on the relationship between CSR and SE performance.

The eighth paper by Bae et al. presented a new theoretical perspective on the relationship between the strength of environmental regulations and foreign direct investment (FDI), which is currently attracting increased scholarly attention. We argue through this new perspective that we should observe the strength of environmental regulations, which has been overlooked by previous literature, and the distance between countries in the FDI relationship. This will make it possible to obtain fresh insights and new ideas regarding the relationship described above.

The ninth paper by Wang and Liu addressed two important research questions: “how may various types of green innovation strategies matter in explaining the variation of firm performance?” and “how may various types of green innovation strategies interact with supply chain risks in illustrating firm performance variation?” Using primary data collected from a sample of 337 firms in China, the study uncovered that the effects of green innovation strategies were statistically significant in elucidating the heterogeneity of firm performance, and their interactions with supply chain risks were also noteworthy and economically important.

The tenth paper by Wang and Teng was motivated to address two major research questions: “what contributes to the development of supply chain management (SCM) capability?” and “how does SCM capability matter in explaining the environmental performance variation of firms operating in large emerging economies?” Using survey data collected from 272 firms in China, the study discovers that specific forms of digital innovation play a positive role in driving the development of SCM capability. The results of the empirical analyses provide further supportive evidence indicating that SCM capability functions as an important role by either partially or fully mediating the relations between the effect of digital innovation and firm environmental performance.

The eleventh paper by Yang and Yang examined the association between dynamic capabilities (DCs) and corporate performance in ESG management. This empirical study tested this as an alternative method using topic modeling with Word2Vec. The findings imply that DCs can enhance corporate performance under uncertainty and pursue a balanced way of sensing-seizing-recofiguring capabilities through ESG management.

The twelfth paper by Son and Kim attempted to examine the relationship between ESG management and financial performance and the role of socially responsible investment by the National Pension Fund (NPF), Korea's largest institutional investor. Their primary discoveries are that Korean firms with good financial performance actively participate in ESG. NPF has a propensity to invest in firms with high financial and ESG performance.

The thirteenth paper by Lee and Yang investigated the impact of CSR performance feedback on future CSR performance to answer the following research questions: First, how does positive CSR performance feedback affect future CSR performance? Second, how does negative CSR performance influence future CSR performance? By performing generalized least squares (GLS) regression analysis based on Korean company data from 2012 to 2019, their empirical findings document that positive social and historical performance feedback positively affected CSR performance.

The fourteenth paper by Liang et al. explored the role of DCs for ESG strategies as well as sustainable management performance. A research model was established by using DC theory, thereby integrating sustainable management and ESG literature. According to the results of their statistical analyses, both absorptive capability and adaptive capability have considerable effects on sustainable management performance through implementation of the ESG strategy as a mediating factor.

The fifteenth paper by Wu and Kuang began with its anticipation that key subordinate executives would play a role linking superiors and subordinates within the top management team (TMT). Based on their recognition of the heterogeneity of TMT preference, data from Chinese listed firms from 2010 to 2019 were used. Their empirical results exhibited a positive relationship between key sub-level executives' governance and accounting conservatism. They also indicated that CEO overconfidence could positively modulate this relationship.

The sixteenth paper by Li and Qamruzzaman examined the nexus of tourism-driven sustainable human capital development (HCD) in emerging economies from 1984 to 2019. In this process, they observed a statistically substantial and long-lasting favorable correlation between tourism and HCD.

The seventeenth paper by Park and Oh identified the different types of information used by investors to make investment decisions based on the UTAUT (Unified Theory of Acceptance and Use of Technology) model. This paper figures out how ESG information affects the decisions of individual investors regarding investments and the factors that affect such behaviors.

The eighteenth paper by Park et al. used text scraped from Twitter. It polished the data to identify trends in ESG themes and their sentimental value over time. This paper informs us how the general public feels about ESG through sentiment analysis.

The nineteenth paper by Lee et al. addressed that ESG at the host country level significantly impacts the implementation of clean development mechanisms (CDM). Moreover, the results of zero-expansion Poisson regression extended the ESG pillar based on institutional theory and emphasized the importance of sustainable development.

As a final remark, the guest editors would like to thank all the reviewers and all those who submitted papers to this Research Topic. The editor-in-chief of Frontiers in Psychology, Axel Cleeremans, also deserves special gratitude. We are incredibly appreciative of the participants' kindness and efforts, both of which have made this special edition possible.

## Author contributions

All authors listed have made a substantial, direct, and intellectual contribution to the work and approved it for publication.

## Funding

This work was supported by the Soonchunhyang University Research Fund.

## Conflict of interest

The authors declare that the research was conducted in the absence of any commercial or financial relationships that could be construed as a potential conflict of interest.

## Publisher's note

All claims expressed in this article are solely those of the authors and do not necessarily represent those of their affiliated organizations, or those of the publisher, the editors and the reviewers. Any product that may be evaluated in this article, or claim that may be made by its manufacturer, is not guaranteed or endorsed by the publisher.
